# Supplementary material for: Are carbon emissions trading and green financial instruments synergistic? -Comprehensive quantitative research based on content analysis
Source: PLoS One. 2024 Mar 7;19(3):e0298601. doi: 10.1371/journal.pone.0298601 (PMC10919601; doi:10.1371/journal.pone.0298601)
Supplement: S1 Data — (ZIP) [file pone.0298601.s001.zip › Data/地级市Z_F工作报告.zip!/║■─╧/╓Ω╓▐╩╨/└┤╫╘╣1⁄2╓┌║┼í╛┬φ┐╦╩2╛▌═°í┐_2005.docx]

各位代表：

　　现在，我代表市人民政府向大会作工作报告，请予审议，并请各位政协委员和其他列席同志提出意见。

　　一、2004年工作简要回顾

　　2004年，我们在市委的正确领导下，深入实践“三个代表”重要思想，努力落实科学发展观，继续突出经济工作三大重点，抢抓机遇，扎实工作，圆满实现了市十二届人大二次会议提出的工作目标，10件实事顺利完成，10件大事取得较大进展，各项主要经济指标增幅创历史新高。

　　——全市实现GDP452.5亿元，比上年增长12.4%；

　　——完成财政总收入33.7亿元，增长21%，其中一般预算收入20.3亿元，增长20.5%；

　　——规模以上工业实现增加值112亿元，增长20%，实现净盈利10.5亿元，增长48.7%;

　　——完成全社会固定资产投资130.3亿元，增长24.3%，达到了三天投资1亿元的水平；

　　——城镇居民人均可支配收入达到10061元，增长14.5%，农民人均纯收入达到3535元，增长16.7%，首次超过城镇居民人均可支配收入增幅。

　　（一）坚持产业主导，经济实力明显增强

　　加快推进产业建设。认真落实中央1号文件精神，加快发展农业和农村经济。全年粮食总产量达到187万吨，特色农产品基地达到35万亩，县域规模以上工业企业发展到380家，实现农产品加工业销售收入37亿元，增长21%；对10万农民进行了实用技术和职业技能培训，农民劳务收入达到54.7亿元；实现农业增加值64.2亿元，增长7.3%。积极推进体制改革和技术创新，大力发展工业经济。在继续深化国企改革的同时，全市完成工业投资41.77亿元，新增生产能力51亿元，实现工业增加值186.1亿元，增长13.4%，完成高新技术产品产值161亿元，增长25%。加快发展第三产业。继续推进芦淞市场群的“两改一提”，中国城、铜锣湾广场等重点项目相继竣工，连锁经营、物流配送等新型商业日趋活跃，旅游、通信、运输、房地产、金融、保险业增势强劲；全年实现社会消费品零售总额159亿元，增长13.6%。同时，大力发展非公有制经济，全年实现增加值198.5亿元，增幅比上年提高1.6个百分点。不断深化各项改革。一是继续大力推进国有企业改革。累计投入改革成本20多亿元，列入改革计划的市属国有企业已有78家基本完成改革任务，其中，粮食流通企业改革经验在全国推广；同时，加大了对在株中央、省属企业改革的支持力度，配合国有大中型企业主辅分离、辅业改制，积极承接企业办社会职能，共接收企业办中小学17所，成功剥离3家企业办医院。二是积极稳妥推进农村各项改革。大力推进农村税费改革，落实粮食直补和良种补贴政策，兑现粮食“两补”资金4311万元，全市农民人平负担减少到27元；茶陵县深化水利设施产权改革的“四换”经验在全省推广，全省农村药品“两网”建设现场交流会在我市召开；农村社会化服务体系不断健全，农民专业合作经济组织达409家；土地征用制度改革和农村信用社体制改革顺利启动。三是因地制宜推进教育体制改革和教育布局调整。推广了炎陵县集中办学经验，进一步优化了教育资源配置。四是大胆探索行政管理体制改革。撤并重组了一批行政机构，开展了事业单位改革试点；启动了财政管理由会计委派、集中核算向国库集中支付改革试点；城市四区区域统计正式铺开。五是积极铺开要素市场改革。全市经营性土地全部实行“招、拍、挂”，共完成土地收益6亿多元；劳动力、人才、资金市场更加活跃，株冶火炬、千金药业成功上市。稳步推进对外开放。继续突出招商引资在对外开放中的核心地位，积极组织参加“欧洽会”、“湘洽会”、“东盟博览会”等经贸活动，加大对招商引资工作的考核和奖惩力度，重奖招商引资中介人，全市实际利用外资1.28亿美元，增长41.9%，实际到位内资45亿元，增长49.8%。全年完成进出口总额5.9亿美元，增长18.7%，其中，出口4.84亿美元，增长32.5%。对外友好往来进一步密切，与我市国际友好城市开展了合作交流；投资450万元购置办公用房，结束了我市驻北京办事处多年来有机构无场所的历史。

　　（二）坚持投入先行，发展平台逐步夯实

　　不断完善城市基础设施。城区控制性详规有序展开，又完成13平方公里旧城改造更新规划。实施“市投区建、区投区建”城市道路建设项目28个，完成投资4.69亿元，建设改造道路37.5公里，其中,建设北路二期、新华东路红旗广场至向阳广场段改造全面完工，庐山路建设接近尾声，株洲大道、金山路正在紧张施工。依法拆除违法建设7.6万平方米，新增公共绿地17.74万平方米，市区绿化覆盖率达38.34%，人均公共绿地面积提高到8.05平方米；石峰、神农、荷塘、天池公园完成计划改造任务。克服重重困难，共完成征地面积7000亩，拆迁城市房屋8.5万平方米，保证了重点工程的顺利实施。解放街等旧城改造项目稳步推进，“烂尾楼”整治已有4栋按计划启动，改造地下管网5.5公里。努力改善农村环境。投资1.46亿元，建设县到乡（镇）公路179公里，完成“通达工程”296公里硬化任务。多元投入办水利的热潮初步形成，水利建设连续三年夺得“芙蓉杯”；全年共投入建设资金5亿多元，加快了小城镇建设，全市城镇化水平达到41.5%，比上年提高0.7个百分点；投入资金1亿多元，建成了一批农业综合开发示范项目；新建1.2万个农村沼气池任务全面完成。积极推进交通能源建设。完成交通基础设施投资9.6亿元，新增等级公路265公里，全市等级公路总里程达到2537公里。浙赣复线电气化铁路和莲易公路株洲段改造有序推进；106国道提质改造完成计划进度。株洲航电枢纽工程累计完成投资12.48亿元；天然气城市管网建设全面铺开；河西220千伏变电站、栗雨110千伏变电站均已进入电气安装阶段；炎陵、茶陵县小水电开发新增装机容量2.34万千瓦。

　　（三）坚持统筹兼顾，社会事业快速发展

　　加快发展各项事业。全市新增高新技术企业26家，10项重大科技成果得到转化，成功开通了全国第二条电动汽车示范营运专线，我市被列为全国五个电动汽车示范城市之一，制造业信息化水平居全省第一。在稳步发展基础教育的同时，积极发展高等教育和职业教育，省中医药学校成功升格为中医药高等专科学校，铁路机械学校、市一职申办高职已通过评审，市三职通过国家级重点中等职业学校评审。网吧、电游室、演出场所存在的突出问题得到集中整治，文化市场进一步规范，文化稽查工作获得全国先进；炎帝节系列活动取得圆满成功，“同一首歌”央视大型演唱会走进株洲，炎帝广场被评为“全国特色文化广场”。全市公共卫生体系框架初步建立，新改建了一批疾控机构、医院传染病区和乡镇卫生院，连续三次被评为“全国无偿献血先进城市”。全民健身和竞技体育运动蓬勃开展。民族工作连续四届获得“全国民族团结进步事业模范集体”。《株洲年鉴（2004）》获全国评比二等奖。广播电视、新闻出版、档案、知识产权和民兵预备役工作都取得新的成效。认真落实三项基本国策。人口与计划生育工作继续保持全省领先地位，人口自然增长率控制在4.86‰，计划生育率达96.7%；妇女儿童事业得到长足发展。环境污染治理力度加大，制定实施了“六条政策”，投入资金1.8亿元，完成污染治理项目141个，依法关停污染严重企业30家，否决不符合国家产业政策、城市发展规划和污染严重的项目55个。通过整治，全年削减废水排放量1100万吨、二氧化硫6500吨、粉尘8000吨。同时，龙泉污水处理厂建设进展顺利，南郊垃圾处理场正式投入使用。国土资源管理坚持保护与开发并举，连续7年实现耕地占补平衡，土地市场和矿产资源管理秩序明显改善。高度关注人民群众生活。重视做好信访接待工作，积极应对、妥善处置了突发群体性事件；狠抓养老、失业、医疗保险扩面清欠工作，共新增参保人数10.85万人，企业基本养老金100%按时足额社会化发放；认真实施《住房公积金管理条例》，全市累计归集公积金12亿多元；新建乡镇敬老院21所；对1200多名农村“五保”老人实行了城市低保户最低生活保障；帮扶6000多名农民脱贫，600多名白内障患者通过“阳光工程”重见光明。

　　（四）坚持职能转变，行政效能不断提高

　　自觉接受监督。认真执行人大及其常委会的决议、决定，自觉接受人大及其常委会的法律监督、人民政协的民主监督和社会各界的监督，全年共办理建议228件、提案260件，答复率、见面率均为100%，满意率99.2%；有2名副市长和9个部门负责人接受了人大述职评议，支持配合人大开展了四部法律的执法检查。加强社会管理。工商、税务、物价、审计、路政、盐政、烟草、海关、质监、招投标、检验检疫等部门大力打击各类不法行为，整顿和治理了市场经济秩序。加强政银企合作，加大贷款清欠和信贷对经济的支持力度，努力创建金融安全区，全年新增贷款59.7亿元。全市荣获驰名、著名商标43件。大力加强社会治安综合治理，有效维护了全市社会稳定。切实加强安全生产监管和消防交通管理，安全生产事故数量、伤亡人数、经济损失分别下降48.8%、51.6%、13.3%，全年没有发生重特大事故。城市管理和交通秩序整治进一步深化，顺利通过了省级文明城市验收。严格依法行政。高度重视政府法制工作，全面贯彻《行政许可法》，各类行政审批事项减少到263项；依法查处各类行政违法案件，有效维护了行政管理秩序。大力开展行政效能监察，努力落实七项工作制度，实行“行政效能监察、经济89110、监察信访举报中心”三位一体的联合办公，全市共查处行政不作为、乱作为案件48起，36个部门和单位接受了行风评议。同时，深入开展廉政建设，切实加大审计监督力度，公务员管理工作得到加强。

　　（五）坚持执政为民，十件实事全面落实

　　着眼于解决与人民群众日常生活密切相关的热点、难点问题，在圆满完成省8件实事分解到我市的42项指标的同时，努力办好年初确定的10件实事：1、支付资金6020万元，对城镇8.24万名“低保”对象全部实行了最低生活保障，对农村2.72万名特困人员给予了每人每月不少于10元的困难补助。2、大力推进城乡安居工程，全年发放住房公积金个人贷款1.82亿元，提取廉租住房建设补助资金200万元，新建拆迁安置房和经济适用房13.6万平方米；1316栋农村特困户安居房全面建成。3、积极做好就业再就业工作，新增城镇就业5.46万人，培训下岗失业人员2.16万人，下岗失业人员再就业3.05万人，新增农村劳动力转移就业6.03万人。4、多渠道投入2550万元，启动了87个社区用房建设，其中57个已交付使用。5、顺利推进了县城电网和农网二期改造，城乡居民生活用电基本实现“同网同价”，国有困难工业企业进行“两电”、“两水”分离的分别为21家、49家，实现用电、用水“一户一表”的家庭分别为20376户、13782户。6、积极建设城市配套设施，16个公厕和5个垃圾中转站已经建成，所有城市公园实行免费开放，市医疗废弃物处置中心建设如期启动。7、大力推进食品药品放心工程，全市农村药品“两网”建设乡镇覆盖率超过70%，蔬菜农药残留超标率低于15%，全市没有发生重大食物中毒事故。8、健全医疗卫生体系，全市137个乡镇建立了独立或相对独立的预防保健站，6所中心卫生院建设已经完工，开展了新型农村合作医疗试点工作；城市社区卫生服务体系建设有序推进。9、制定实施了《株洲市中小学贫困学生救助办法》，市县两级共筹集资金500多万元，救助城乡贫困学生1.35万人；进城务工人员子女享受同等教育权利政策在秋季全面执行；投入2000多万元加快了教育信息化建设，全市所有中学和城市小学以及80%以上的农村中心小学拥有电脑教室。10、积极开展“平安株洲”创建工作，110、119、122“三台合一”整合试点工作已经启动，全年共破获各类刑事案件11642起，查处各类治安案件16638起，现行命案侦破率88.7%；消防特勤站已动工建设。同时，本届政府所列的十件大事也取得较大进展：1、《株洲老工业基地改造与振兴规划纲要》已上报国家发改委。2、产业规划指导作用增强，六大支柱产业、三大新兴产业发展加快，全市产值过亿元的企业增加到51家，其中过5亿元的14家，过10亿元的7家。3、全市投入农田水利建设资金3.87亿元，兴修了一批水利工程，旱涝保收面积提高到85%，加快了市区4个防洪闭合圈建设；洮水水库已经正式开工。4、醴潭高速启动建设，衡炎、长株高速今年有望动工；炎帝公路、沔桃公路改造已经完成；武广铁路客运专线已经确定在株洲设站。5、城市快速环道累计完成投资13.2亿元，东湖立交竣工通车，建宁大桥、西环线、南环线建设进展顺利；湘江四桥、五桥和景观防洪堤建设前期工作基本完成。6、严格控制重点污染源，城市生活污水处理率、生活垃圾无害化处理率分别达到51.06%和91%，饮用水源水质达标率稳定在96%以上；清水塘工业区环境综合治理首批5个项目可望争取国家1亿元资金支持；建宁港、白石港和天鹅湖治理已经或即将动工；超额完成了80多万亩造林、抚育、低改任务，其中退耕还林18.8万亩，全市森林覆盖率达56.9%；投入1100多万元，完成水土流失面上治理100平方公里。7、湖南工业大学（筹）正式挂牌，已有3000多名学生在河西新校区就读。8、市中医院门诊大楼已封顶，河西医院征地拆迁接近尾声。9、总投资4亿元的市体育中心主体工程顺利推进，累计完成投资3亿元。10、文化园三期建设、株洲影剧院改造工程正在招商引资，炎帝陵二期工程建设规划已经审定。

　　各位代表，过去的一年，在宏观经济环境偏紧的形势下，我们取得的成绩比预期的好。

　　这是市委正确领导，市人大、市政协支持监督和全市人民团结一心、奋力拼搏的结果。在此，我代表市人民政府，向全市广大工人、农民、知识分子、干部职工、驻株部队和武警官兵，向关心和支持株洲建设的各民主党派、人民团体以及社会各界人士，表示崇高的敬意和衷心的感谢！

　　同时，我市经济社会发展中还存在不少困难和问题，主要是：改革任务艰巨，影响加快发展的体制性、机制性矛盾依然突出；投入仍然不足，电力、土地等能源、资源因素对经济增长的制约逐步增大；社会管理相对滞后，维稳压力较大，环境污染仍需大力治理，城市管理水平有待提高；少数公务员服务意识还不强，工作效率和服务水平还不高；等等。对于这些问题，我们将进一步采取有效措施，切实加以解决。

　　二、2005年工作基本设想

　　2005年，是“十五”计划的最后一年，也是我市经济社会发展充满机遇与希望的一年。纵观国内外形势，以欧美日为主的世界经济稳步回升，我国对外开放向内地梯度推进，中部崛起战略正式实施，长株潭经济一体化进程不断加快，这些都为我们加快发展提供了难得机遇。我们一定要充分利用各种有利条件，紧扣市十二届人大一次会议确定的本届政府任期目标，认真贯彻市委九届十二次全体会议精神，贯彻落实科学发展观，继续坚持“三个第一”，突出经济工作三大重点，加快推进“三化”进程，努力促进经济较快发展和社会全面进步，确保全面实现“十五”各项工作目标，为“十一五”期间更快更好发展奠定坚实基础。全市经济社会发展的主要预期目标是：GDP比上年增长11.5%；全社会固定资产投资增长20%；财政总收入和一般预算收入均增长12%；城镇居民人均可支配收入增长10.5%，农民人均纯收入增长5%；城镇登记失业率控制在4.6%以内，人口自然增长率控制在6.5‰以内；城市化水平提高到42%；各项事业都有新的进步。

　　（一）突出产业建设，提高发展水平

　　加快发展，在任何时候、任何条件下都必须紧紧扭住产业这个关键，加大项目开发力度，拓宽产业投入渠道，提升经济发展水平。做强工业经济。株洲靠工业立市，做强工业经济，我们将立足现有基础，坚持以信息化带动工业化，工业化推动信息化，积极发展循环经济，努力走出一条科技含量高、资源消耗低、环境污染少、人力资源优势得到充分发挥的新型工业化道路。坚持规划引导，壮大支柱产业。充分发挥市场基础作用和企业主体作用，抓好产业发展规划和产业政策的实施，巩固壮大交通装备制造、有色冶金及深加工、农产品加工、化工、陶瓷等支柱产业。突出改造提升，打造品牌企业。支持企业运用高新技术和先进适用技术改造提升传统产业，支持株硬集团钨钼深加工、株冶集团锌系统节能降耗、智成化工11.5万吨双氧水、株化集团年产20万吨PVC、太子奶公司乳品和食品工程、南方公司中小航空发动机基地等重大项目建设，全年力争完成新上工业项目和技改投入50亿元。通过改造提升，加快形成一批核心竞争力强、主业突出、拥有自主知识产权和知名品牌的大公司、大集团。加快园区建设，培育产业集群。继续加大政策扶持力度，全力支持高新区、醴陵陶瓷科技园等工业园区的基础设施建设；立足园区产业定位，加大项目进园力度，积极引导和支持企业向园区聚集，把工业园区办成产业发展的聚集区。做大县域经济。一是切实加快农村“三化”进程。坚持用工业化的理念指导农业和农村经济发展，充分依托资源优势，发展县域工业和农产品加工业；大力推进农业产业化，加大农业结构调整和农产品基地建设力度，继续围绕竹木、肉食、油脂、果蔬、优质米等优势产业，支持壮大龙头企业，拉长产业链条，进一步提高农产品加工企业对结构调整和农民增收的带动能力；加快推进农村城镇化进程，努力构建企业、市场、小城镇“三位一体”的发展格局，引导小城镇走产业兴镇、产业强镇之路。二是全面落实各项支农政策。深入贯彻中央1号文件，落实农村税费改革各项政策，巩固改革成果；落实基本农田保护政策，进一步稳定和完善家庭联产承包责任制；构建多元投入体系，拓宽农业投入渠道。三是不断完善农村服务网络。进一步完善农村市场体系，积极发展农村专业合作经济组织；创新农业技术推广体系，切实做好农业病虫害和动物重大疫病防治工作；改善金融服务，鼓励农村信用社增加农户小额信用贷款和农户连保贷款。四是积极发展非农经济。加大对农民工的培训力度，提高劳务输出组织化程度，扩大劳务输出规模。做优第三产业。在科学制定市区商业网点规划的基础上，按照“政府引导、民间投资”的方针，继续抓好芦淞等市场的“两改一提”，支持家润多商业广场、建宁购物公园、杉木塘生产资料大市场、金属材料市场、中南服饰会展中心等一批重点项目建设；鼓励商贸流通企业推行连锁经营、电子商务等经营业态，逐步推进“农改超”，增强市场竞争能力。加大中国优秀旅游城市创建工作力度，进一步提升旅游景区（点）档次，做大做强以炎帝陵为龙头的旅游品牌，大力推介湘东旅游精品线路，加快发展旅游业；抓紧长株潭现代物流中心、中南农产品物流中心和芦淞服饰物流仓储配送中心等项目建设，整顿规范芦淞市场群物流配送秩序，大力发展现代物流业；坚持市场导向，合理引导普通住宅建设，适当控制商业地产和高档商品房投资规模，搞活住房二级市场，支持发展房地产业；根据国家相关政策，进一步支持金融、保险、电信等服务业的改革，规范发展律师、会计、公证、咨询、担保等中介服务业。

　　无论是做强工业，做大县域经济，还是做优第三产业，都必须以增加投入为基础。没有固定资产投资的大幅增长，没有一批建设项目作支撑，就不可能有新的经济增长点和新兴产业的形成，也不可能有经济社会发展的后劲。加大固定资产投入，我们将重点把握好这几个环节：一是认真策划、包装好项目，切实增强招商引资和争取中央、省里支持的针对性；二是全力支持企业加大技改投入，加快在建项目的实施进度；三是加强政银企合作，推动金融机构增加信贷投放；四是制定实施投资奖励政策，建立投资促进机制，进一步激活民间投资。

　　（二）突出改革开放，增强发展活力

　　实践证明，没有改革的大突破，就不可能有经济的大发展；没有开放的大推进，就不可能有经济增长的大跨越。深化改革，扩大开放，发展非公有制经济，在很多领域内、很多层面上，都互相促进，紧密相关。继续深化各项改革。一是国有企业改革。加大市属国有企业改革攻坚力度，力争年内基本完成改革任务；积极支持在株中央、省属国有企业改革；鼓励支持中小企业购并重组，积极推动符合条件的企业上市融资；深化国有资产管理体制改革，进一步完善国有资产授权经营制度，依法强化对国有资产的监管，确保国有资产保值增值。二是农村各项改革。深化农村税费改革，根据国家有关政策和省政府的部署，减免农业税；加快乡村消赤减债步伐；按照依法、自愿、有偿的原则，引导并规范农村土地使用权流转。三是投融资体制改革。切实推进投资主体多元化，确立企业投资的主体地位，进一步减少、改进和规范投资审批；缩减财政投资的范围和领域，健全政府投资项目法人约束机制。四是行政管理体制改革。进一步精简审批事项，减少审批环节，创新行政许可体制和机制；改革和理顺财政管理体制，完善预决算制度和国库集中支付制度，健全公共财政体制；坚持政事分开，年内全面启动事业单位改革；积极稳妥推进乡镇和街道办事处的区划调整。五是要素市场改革。不断完善土地、技术、人力资源等要素市场，大力发展资本市场；按照“区别对待、分类指导”的原则，基本完成农村信用社体制改革。全面扩大对外开放。加大招商引资力度。转变招商引资主体，营造政府引导、企业为主、全民招商的浓厚氛围。创新招商引资方式，大力推行以商招商、代理招商、网上招商、园区招商、派出小分队“上门”招商等多种招商形式。完善招商引资工作机制，建立重大项目市县级领导带头招商和联系协调制度，完善招商引资目标考核、奖励、跟踪落实办法，营造“亲商、安商、富商”的环境，不断提升招商引资的层次和水平。积极拓展国际国内市场，鼓励、支持中小企业和民营企业自营出口，鼓励有条件的企业“走出去”，建立海外资源和生产基地。大力发展非公有制经济。依法维护非公有制经济的平等竞争地位，清理取消一切有碍于非公有制经济发展的政策规定。在市场准入、土地使用、税收、融资等方面，加大对非公有制经济的支持力度，鼓励民营企业参与国有企业改革、城乡基础设施建设和农业产业化经营。鼓励和帮助非公有制企业做好上市申报工作，直接从资本市场融资。引导非公有制企业依法经营，照章纳税，诚实守信，自觉维护企业职工的合法权益，保障职工切身利益。

　　（三）突出以人为本，构建和谐社会

　　发展，是经济与社会的协调发展，是人与自然的和谐发展。加快株洲发展，必须贯彻落实科学发展观，以更加有力的措施维护社会稳定，以更加有效的途径发展各项事业，以更加饱满的热情关注民情民生。全力维护社会稳定。稳定是深化改革、加快发展的前提条件，是人民群众安居乐业的基本保障。维护社会稳定，我们将重点做好四个方面的工作：一是继续开展“四五”普法活动，提高市民法律意识，积极引导群众运用法律武器、在法律的框架内化解矛盾，协调关系；二是高度重视、认真调处人民内部矛盾，进一步改进信访接待工作，妥善处置群体性事件；三是切实加大社会治安综合治理力度，积极开展“平安株洲”创建活动，坚持“打防结合，预防为主”的方针，开展“严打”和专项整治斗争，特别是对严重暴力犯罪、有组织犯罪、侵财性犯罪和破坏公共设施的犯罪，要重点防范、重点部署、重拳出击，切实增强广大人民群众的安全感。同时，认真抓好隐蔽战线工作，进一步加强交通、消防和生产安全监管，防止发生重特大安全事故。大力发展各项社会事业。进一步完善科技创新体系，努力建设高素质科技人才队伍，提高科技引领水平。继续改革基础教育管理体制，稳步推进中小学布局调整，积极发展民办教育，结合剥离企业办学校，建立多渠道投入的社会办学机制；继续支持湖南工业大学建设，做大做强高等教育和职业教育，努力开创各层次教育蓬勃发展的良好局面。加大城区医疗体制改革力度，加强卫生执法工作，整顿和规范卫生医疗市场秩序，加快社区卫生服务体系建设，建成市区16个社区卫生服务中心和83个服务站。建立网吧监管平台，加大网吧清理整治力度，新建改建一批文化场馆（站），继续举办好“周周乐”等群众文化活动，鼓励创作，力出精品。大力发展广播电视文化产业，逐步实现城区数字电视整体平移。全力办好市十运会，积极备战省十运会，力争市体育中心主体工程年内竣工。同时，重视和做好新闻出版、民族宗教、人防、档案、地方志、残联等工作。坚持走可持续发展道路。全面落实人口与计划生育目标管理责任制，稳定低生育水平，提高人口素质。认真实施2001—2010年妇女、儿童发展规划，推动妇女儿童事业发展跃上新台阶。切实加强土地管理，严格落实耕地保护制度，大力整顿和规范土地、矿产市场秩序。以清水塘工业区环境综合治理为重点，积极开展“蓝天行动”，加强环境保护，务必站在全市长远发展的高度，站在保障市民身体健康的高度，站在造福子孙后代的高度，宁可GDP少增长几个百分点，宁可其他方面过点紧日子，也要千方百计加大污染治理力度，把一个环境优美的株洲献给市民，留给子孙，还给自然。努力为民办好十件实事。1、加强城乡社会保障工作。依法做好企业基本养老保险、失业保险、医疗保险扩面征缴工作；城镇“低保”标准由156元提高到170元；帮扶6200名农村贫困居民脱贫，农村特困户救济率100%；改扩建敬老院20所，集中“供养”五保户增加到2000人。2、推进就业和再就业。新增城镇就业4.5万人，下岗失业人员再就业2万人；培训下岗失业人员1.5万人，农业实用技术培训10万人次、农民职业技能培训2万人次，新增农村劳动力转移就业5万人。3、继续推进农村中小学危房改造。完成危房改造面积4.3万平方米；健全中小学贫困生救助机制。4、大力改善城市环境。重点治理废气污染，全年市区空气良好天数增加15天以上；启动锅炉改造工程，逐步推行“煤改气”；新增社区、街头绿地15处以上。5、全面实行农村计划生育奖励扶助政策。让符合政策的6000个左右对象得到奖励扶助。6、加强农村基础设施建设。全面完成国家计划内县到乡镇公路建设，继续推进“通达工程”；新建农村沼气池6000个。7、努力缓解中心城区交通拥挤问题。合理布局城区公共交通站场，改造、提升分流交通的城区支路15条，对地下停车场违规改建商场进行全面清理，实行还场（地）于车。8、改善城乡中低收入居民居住条件。适度建设经济适用住房，建立廉租住房制度，新增领取廉租住房补贴的家庭300户；发放住房公积金个人贷款1亿元以上。9、加大社区建设力度。重点完善社区服务功能，改扩建40栋社区服务办公用房。10、继续实施食品药品放心工程。全面完成农村药品“两网”建设，加强食品生产流通环节的监管；改造14所乡镇卫生院，搞好农村新型合作医疗试点。

　　（四）突出环境建设，争创发展优势

　　环境就是生产力，抓环境就是抓发展。我们将继续坚持把优化环境作为政府的第一责任，在努力抓好基础设施等“硬”环境建设的同时，下大力气整治好“软”环境。加强基础设施建设。按照“东提西拓、合拢三角”的城市建设方针和土地利用总体规划修编、第六次城市总体规划要求，继续全面推进城乡基础设施和交通能源建设。城区方面，集中财力加快建成建宁大桥、东环A段、红旗立交、京渌立交、省道S211立交，完成西环线、南环线、东环线路基工程，实现城市快速环道路基全线贯通、株洲大道以及栗雨工业园环线建成通车；抓好湘江四桥、湘江防洪景观堤建设，加快治理建宁港、白石港和天鹅湖；全面完成市区4个防洪闭合圈建设；龙泉污水处理厂建成投产；在国土政策许可的前提下，通过市场运作，拉通黄河北路、修建新塘路、改造省道S211（城区至快速环道南环线段）；采用“区建市补”的方式，拉通、改造、提升南华路、育才路、上月塘路、建设巷、天平路、太平路、公园西路等15条城区支路;继续加快红旗广场、响石广场、火车站广场等区域旧城改造;围绕创建国家园林城市目标，开展“全民造绿”，提高城市主次干道绿化水平，提升庭院小区园林档次，在改造提升现有城市公园的基础上，启动河西天台公园建设。农村方面，加快农田水利基本建设，继续实施“十百千万”工程；力争农业综合开发投入达到1亿元，高标准改造一批中低产田和灌区水利骨干工程；加强农业生态环境建设，做好林地林权登记换发证和43万亩退耕还林管护工作。交通方面，按照“建设主骨架、改善干支网，提高等级率，扩大覆盖面”的思路，支持加快醴潭高速公路建设，力争衡炎、长株高速公路和亭网（朱亭至网岭）公路启动建设；完成106国道醴陵至炎陵县城全线提升改造工程；做好伞铺至醴陵、省道S320（茶陵县城至界化垅段）公路改造的前期工作；提高县乡公路等级率5个百分点。能源方面，积极推进县城电网改造工程，加快建设向阳村110千伏变电站，完成市区莲花、醴陵浦口等5个110千伏变电站以及攸县大塘冲220千伏变电站扩建工程；支持加快株洲航电枢纽、洮水水库建设；加快实施天然气入株工程，确保年内实现城区供气。优化发展“软”环境。一是不断优化政务环境。启动“电子政务”工程，继续加强政务服务中心建设，严格实行“一个窗口受理、一站式收费”，大力推进政务公开；继续开展行政效能监察，全面落实以办事时限制为重点的“七项制度”，充分发挥“经济89110”的作用，严肃查处破坏经济发展的行为。二是不断优化市场环境。充分发挥各有关部门的职能作用，加强市场监管，大力打击各类欺行霸市、制假售假、偷税漏税、违规金融、贩黄贩非、无证经营等不法行为，努力营造公平有序的市场环境；加强社会诚信体系建设，支持银行清欠个人不良贷款，营造良好的金融环境。三是不断优化建设施工环境。严格依法依规做好征地拆迁工作，切实保护业主合法权益，严厉打击少数围攻谩骂执法人员、阻工“敲竹杆”的不法分子。四是进一步改善城市生活环境。以相对集中城市管理执法权为契机，完善城市管理网络，下移管理重心，提高城市管理水平；以整治环境卫生、市容市貌和公共客运秩序为重点，大力改善城市环境。（五）突出依法行政，加强自身建设坚持以建设“有限政府、责任政府、服务政府、法治政府”为目标，切实抓好政府自身建设，不断提高依法行政的水平、科学决策的能力，树立勤政廉政的良好形象。不断提高依法行政水平。继续深入贯彻《行政许可法》，改革和理顺行政执法体制，加强政府执法监督，切实提高政府执法水平。继续推进政府职能转变，主要运用经济手段和法律手段管理经济，着力解决政府职能“越位”、“缺位”和“错位”的问题。努力坚持民主科学决策。进一步完善重大事项定期向人大报告、向政协通报制度，认真落实人大及其常委会的决议、决定，积极办理人民代表的建议和政协委员的提案，多渠道听取各民主党派、工商联、无党派人士、人民团体和社会各界的意见。建立和完善专家咨询、社会公示和听证制度，逐步完善专家论证、公众参与和政府决策相结合的决策机制。切实加强勤政廉政建设。全面落实党风廉政建设责任制，积极运用行政监察和审计监督手段，突出抓好各级领导干部的廉洁自律，重点纠正部门和行业不正之风。严格要求，严格管理，努力建设一支廉洁奉公、勤政为民、业务精通、作风优良的公务员队伍。

　　各位代表、同志们，抢抓机遇，时不我待；加快发展，只争朝夕。让我们在市委的正确领导下，高举邓小平理论伟大旗帜，努力践行“三个代表”重要思想，把崇高的使命化为积极的行动，同心同德，开拓进取，为建设更加美好的株洲而努力奋斗！
